# Lenalidomide ameliorates cognitive impairment via putative AChE inhibition: an in silico and in vivo study in a scopolamine-induced cognitive impairment model

**DOI:** 10.1038/s41598-026-49461-8

**Published:** 2026-05-23

**Authors:** Salehin Sheikh, Md. Shadin, Tanzila Akter Eity, Most. Israt Jahan Oni, Mst Muslima Khatun, Raihan Chowdhury, Md. Shimul Bhuia, Mohammed Alfaifi, Faisal H. Altemani, Abdullah H. Altemani, Na’il Saleh, Muhammad Torequl Islam

**Affiliations:** 1 Department of Pharmacy, Gopalganj Science and Technology University, Gopalganj, 8105 Bangladesh; 2 Bioinformatics and Drug Innovation Laboratory, Bioluster Research Center Ltd., Gopalganj, Dhaka, 8100 Bangladesh; 3 Department of Clinical Laboratory Sciences, College of Applied Medical Sciences, King Khalid University, Abha, 61421 Saudi Arabia; 4 Department of Medical Laboratory Technology, Faculty of Applied Medical Sciences, University of Tabuk, Tabuk, 71491 Saudi Arabia; 5 Department of Family and Community Medicine, Faculty of Medicine, University of Tabuk, Tabuk, 71491 Saudi Arabia; 6 Pharmacy Discipline, Khulna University, Khulna, 9208 Bangladesh; 7 Department of Chemistry, College of Science, United Arab Emirates University, POBox 15551, Al Ain, United Arab Emirates

**Keywords:** Acetylcholinesterase inhibitors, Cognitive impairment, Lenalidomide, Neurodegenerative disease, Molecular dynamics, Computational biology and bioinformatics, Drug discovery, Neuroscience

## Abstract

Alzheimer’s disease (AD) causes progressive cognitive decline, and current therapies provide limited benefit. This study evaluated the neuroprotective effects of lenalidomide (LLM), a thalidomide derivative, in a scopolamine-induced mouse model of cognitive impairment, with emphasis on its acetylcholinesterase (AChE) inhibitory potential. Mice received LLM (5, 10, and 20 mg/kg), donepezil (DNP) (3 mg/kg), or a combination and were assessed using Y-maze, passive avoidance, novel object recognition, and Morris water maze tests. In silico analysis, including molecular docking, 100 ns molecular dynamics simulation and ADMET profiling were performed to investigate the interaction of LLM with AChE. Memory performance showed a significant and dose-dependent improvement after the treatment of LLM. The 20 mg/kg dose exhibited effects comparable to DNP. LLM and DNP work together to increase effectiveness. Docking and simulation analyses revealed strong, stable binding to AChE while ADMET values indicated good drug-likeness. LLM exhibits neuroprotective and cognition enhancing effects in the scopolamine-induced model. In silico study also shows its potential as an AChE inhibitor. The study’s anti-inflammatory mechanisms might also be helpful but need more exploration.

## Introduction

Alzheimer’s disease (AD) is typically manifested as an insidious memory loss, eventually accompanied or followed by other cognitive deficits such as visuospatial and navigation difficulties, as well as executive dysfunction and language impairment. Many cognitive impairments affect normal activities in daily lives, while the onset of the disease usually comes with several behavioral and psychological symptoms of dementia (BPSD)^1^. It is estimated that 131 million people will have AD dementia by 2050 which will cause substantial impact on the world. The need for preventive strategies to postpone the onset, retard the progression and mitigate the symptoms of AD is urgent^2^.

According to cholinergic theory AChE is responsible for memory dysfunction and cognitive decline due to depletion of ACh in the synaptic cleft^3–5^. AChE is crucial to cholinergic hypothesis of AD. In AD, there is a markedly increased activity of AChE which leads to the accelerated hydrolysis of ACh and this impairs cholinergic neurotransmission in brain regions whose cholinergic in nature and are responsible for learning and memory like the hippocampus and cortex^6^. Therefore, pharmacological interference with AChE remains one of the best validated approaches to the treatment of AD, confirmed by clinically used drugs like donepezil (DNP) rivastigmine and galantamine. Latest preclinical studies have corroborated AChE as a prospective target for natural and synthetic compounds, establishing AChE inhibitors not only as agents to restore cholinergic tone but also to attenuate oxidative stress, neuroinflammation, and amyloid-β aggregation^7–9^. That is, a central goal of AD drug discovery must remain the identification of novel AChE inhibitors with multimodal neuroprotective potential.

Accordingly, pharmacological inhibition of AChE remains a validated symptomatic strategy, as evidenced by FDA-approved drugs such as DNP, rivastigmine, and galantamine. However, their long-term clinical utility is limited by adverse effects, including hepatotoxicity^10^.

Natural products have been researched and used for treating disease from the beginning of human history^11^. Since natural products have few adverse effects, reliable safety records, and are cost-effective, their usage as medicinal compounds extracted from fungi, plants, and herbs is considered an essential precursor to modern medicine^12^. The discovery of numerous natural compounds from plant extracts with acetylcholinesterase (AChE) inhibitory activity has been shown to enhance cholinergic function and alleviate symptoms associated with cholinergic dysfunction^13^.

Lenalidomide (LLM), a derivative of thalidomide also called immunomodulating agents (IMiDs), showed diverse pharmacological activities, including antiemetic, antiinflammatory, antiapoptotic, antioxidant, anticancer, neuroprotective effects, and antiangiogenesis^14,]^^15,]^^16,]^^17^. In addition to inhibiting Akt phosphorylation at Ser473 and Thr308, LLM is known to downregulate important prosurvival cytokines like tumor necrosis factor-α (TNF-α), interleukin-6 (IL-6), interleukin-8 (IL-8), and vascular endothelial growth factor (VEGF). It also activates immune effector cells (T and NK cells), stimulates T-cell proliferation, and increases production of IL-2 and interferon (IFN)-γ through T-cell receptor activation, and proliferation of NK cells^16^. The neurological adverse effects of thalidomide, such as drowsiness and neuropathy, are absent from LLM, which allows for long-term administration and is used to treat neurodegenerative illnesses like Parkinson’s disease and conditions involving cholinergic deficiency such as, AD. LLM also has a predictable tolerability profile^18^.

Beyond its well-characterized immunomodulatory and anti-inflammatory properties, lenalidomide has shown neuroprotective efficacy in preclinical models of neurodegeneration, such as Parkinson’s disease, where it reduced microglial activation and improved motor function^17^. Although a direct interaction between LLM and cholinergic targets has not been previously established, its structural resemblance to thalidomide a molecule known for its ability to engage diverse biological targets along with its favorable pharmacokinetic profile, including central nervous system penetration^18^, led us to hypothesize that LLM might also influence cholinergic neurotransmission. Given the centrality of AChE inhibition in alleviating cognitive deficits and the growing interest in multi-target agents for AD, we postulated that LLM could potentially act as an AChE inhibitor, thereby combining cholinergic enhancement with anti-inflammatory activity.

The current study aimed to evaluate the AChE inhibitory potential of LLM in a scopolamine-induced cognitive impairment model. Simultaneously, an in silico investigation was conducted to characterize the molecular interactions of LLM with AChE and to assess its pharmacokinetic and toxicological profiles in the context of neurotherapeutic development.

## Materials and methods

### Chemicals and reagents

LLM was purchased from Chengdu Alfa Biotechnology Co., Ltd. (China), CAS No: 191732–72-6. Scopolamine (SCP), DNP, and other materials were obtained from normal commercial sources and were of the highest grade available.

### Dose selection

The test doses of LLM (5, 10, and 20 mg/kg, i.p.) were selected based on previously published literature where LLM demonstrated neuroprotective and anti-seizure effects in rodent models^19^. The positive control drug, DNP was administered at 3 mg/kg (p.o.), a dose established in prior AD preclinical studies for its cognitive-enhancing and neuroprotective efficacy^20^. Scopolamine (SCP) was administered at 3 mg/kg (i.p.), a standard dose widely used to induce reliable and reproducible cholinergic dysfunction and cognitive impairment in rodent models of AD^21^^[,22^. The control group received the vehicle (normal saline containing 0.02% DMSO) at 10 mL/kg. No pilot dosing was performed, as the selected doses are well-established in the literature for the respective compounds in similar cognitive impairment paradigms.

### Experimental animals

Male *Swiss albino* mice (18–22 g, 2.5–3 months old) were obtained from Jahangirnagar University, Savar, Bangladesh. The animals were housed in sterile plastic cages (290 × 220 × 140 mm, 5 mice per cage) under standard conditions: temperature 26 ± 2 °C, humidity 65%, 12 h light/dark cycle, with ad libitum access to food and water. Mice were acclimatized for seven days prior to experimentation. All procedures were approved by the GSTU Animal Ethics Committee (Approval No.: #GSTU-pharmacy-20phr001; Date: August 1, 2025) and conducted in accordance with institutional guidelines and the ARRIVE guidelines Percie^23^. To avoid potential carry-over effects, stress, or learned behavior from one test influencing the results of another, a separate cohort of mice was used for each of the primary behavioral tests (Y-maze, novel object recognition, passive avoidance, and Morris water maze).

### Randomization and blinding

Animals were initially assessed for general health status, body weight, and basic locomotor activity to ensure suitability for behavioral testing. Pre-screening for learning capability was strictly limited to excluding animals with evident sensory or motor impairments that could interfere with task performance, without any ranking based on cognitive ability, thereby preserving a baseline physiological state across the study population.

Following this, mice were randomly assigned to seven experimental groups (n = 5) using a computer-generated random number sequence to eliminate selection bias. Group allocation was concealed from the experimenters, and all treatments were coded to maintain blinding. Both behavioral assessments and data analyses were conducted by investigators blinded to group assignments, with treatment codes revealed only after completion of statistical analysis to ensure unbiased outcome evaluation.

### Exclusion criteria

Animals were excluded from analysis if they exhibited:

- Illness or injury unrelated to treatment
- Failure to explore in behavioral tests (e.g., no movement in Y-maze or NOR)
- Extreme outliers in behavioral performance (defined as values beyond mean ± 3 SD)
- No animals were excluded from the study based on these criteria.

### Sample size and allocation

A total of 140 mice were used in this study and divided into four independent cohorts (35 mice per cohort), each assigned to a single behavioral paradigm (Y-maze, Novel Object Recognition, Passive Avoidance, or Morris Water Maze) to eliminate carryover effects associated with repeated testing. Within each cohort, animals were randomly allocated into seven groups (n = 5).

The sample size was determined based on statistical considerations and validated through post-hoc power analysis using GPower 3.1 software. The observed effect sizes (Cohen’s f = 1.39–3.40) across behavioral assays yielded a statistical power (1 − β) of > 0.999 at a significance level of α = 0.05. Although a sample size of n = 6 is commonly used in behavioral studies, our analysis confirmed that n = 5 per group was sufficient to reliably detect the large effect sizes associated with scopolamine-induced cognitive impairment and treatment effects. Furthermore, power estimation indicated that a minimum of 3–5 animals per group would be adequate to achieve the conventional 80% power threshold (1 − β = 0.80).

The use of multiple behavioral paradigms was intended to assess distinct cognitive domains, including spatial working memory (Y-maze), recognition memory (Novel Object Recognition), associative learning (Passive Avoidance), and spatial reference memory (Morris Water Maze). To ensure unbiased outcomes and minimize stress, fatigue, or learning interference, no animal was subjected to more than one behavioral test. For paradigms involving multiple phases (e.g., training and probe sessions in MWM and NOR), a minimum interval of 24 h was maintained between sessions to allow adequate recovery and ensure reliable performance.

### Animal euthanasia

Mice were euthanized humanely in a chamber filled with 100% compressed carbon dioxide (CO_2_) at a flow rate that displaced 20% of the volume of the chamber per minute. The cessation of respiration was confirmed dead by cervical dislocation. All processes were sanctioned by the GSTU Animal Ethics Committee and fall within the ambit of the AVMA Guidelines for the Euthanasia of Animals.

### Experimental design

**Fig. 1:**
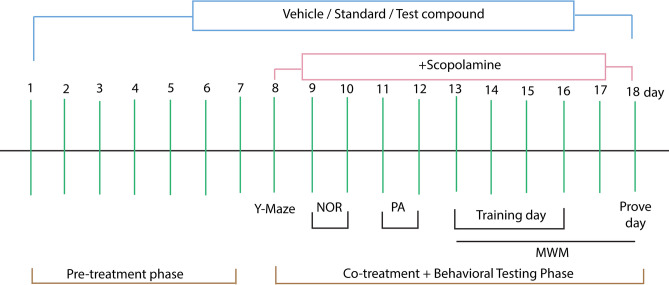
Schematic representation of the experimental outline [NOR: Novel object recognition; PA: Passive avoidance; MWM: Morris water maze;] Note: The 18-day experimental period was optimized to include a 7-day drug pre-treatment phase followed by a 10-day behavioral assessment window. This duration ensures the establishment of stable pharmacological levels before inducing cognitive impairment and is consistent with sub-acute neuroprotection models.

After a seven-day acclimatization period under standard laboratory conditions, *Swiss albino* mice were randomly divided into seven groups (n = 5): Group I (Normal) (N) received saline containing 0.02% dimethyl sulfoxide (DMSO); Group II (Negative Control) received SCP (3 mg/kg, i.p.) to induce cholinergic dysfunction and cognitive impairment; Group III (Standard Treatment) received DNP (3 mg/kg; p.o.) followed by SCP; Groups IV, V, and VI were treated with LLM at doses of 5, 10, and 20 mg/kg (i.p.), respectively, each in combination with SCP; and Group VII received a combination of high-dose LLM (20 mg/kg; i.p.) and DNP (3 mg/kg; p.o.) followed by SCP to assess potential synergistic effects. From day 1 to day 7, all groups received daily oral or intraperitoneal treatments of their respective test compounds or vehicle. From day 8 to day 14, SCP was administered intraperitoneally 30 min after oral or intraperitoneal dosing in all groups except the N. Behavioral testing was performed 30 min after SCP injection. The Y-maze test was conducted on day 8. The novel object recognition test on days 9 and 10 (familiarization and testing phases), The passive avoidance test on days 11 and 12 (training and retention phases), Morris water maze test from days 13 to 18. The probe test was done one day after the training phase (training: Days 13–16; probe test: Day 18). The experimental design is in detailed in Fig. 1. All experimental procedures were conducted under institutional ethical guidelines for the care and use of laboratory animals.

### In vivo behavioral study

#### Y-maze test

The maze was composed of three wooden arms that formed a "Y" configuration when joined in the center. The mouse could view distal spatial features because of the arms’ 10 cm-high walls. A previously published protocol served as the basis for the Y-maze drawing and technique, which were modified to fit the mouse system^24^. The measurements (120^◦^; 35 cm long, 7 cm wide, and 10 cm high) are uniformly spaced. In a Y-maze, spontaneous alternation performance was evaluated. The rodents were allowed to roam freely for five minutes after being placed in one of the branch chambers. A manual recording of the arm entry sequence was made. Mice were given LLM at different doses (5, 10, and 20 mg/kg; i.p.) an hour prior to the test, along with DNP (3 mg/kg; p.o.) as a positive control. Thirty minutes later, SCP (3 mg/kg; i.p.) was administered to cause memory impairment. Animals in the control group were given a 0.02% (DMSO) solution in place of LLM. In between testing, 70% ethyl alcohol was used to clean down the Y-maze. “Alternation” was described as the capacity to move to all three arms simultaneously. The alternation score for each mouse was defined as the ratio of the actual number of alternations to the possible number (defined as the total number of arm entries minus two) multiplied by 100, as shown by the following equation: Alternation (%) = [(Number of alternations)/(Total arm entries − 2)] × 100. The number of arm entries per trial was used as an indicator of locomotor activity.

$$\text{Spontaneous Alternation Behavior }(\mathrm{\%}) =\left(\frac{\text{Number of Alterations}}{\text{Total entries in arms}-2}\right)\times 100$$

#### Novel object recognition

The novel item recognition test measured the amount of time mice spent investigating both new and unfamiliar things. With a few slight adjustments, this approach is comparable to that of Ennaceur and Delacour et al. (1988). Two identical objects, A1 and A2, were placed in a relative arrangement in a well-lit testing arena (40 × 40 × 30 cm). Mice were inserted into the apparatus back-to-back and allowed to explore for five minutes before being removed. After 24 h, one of the identical items was replaced with the new object B. After the mouse was brought back into the arena, the number of times it engaged with the new item B over the course of five minutes was tracked. Timely cleaning of objects and the experimental instrument was necessary to reduce the impact of odor.

$$\text{Discrimination index}= \frac{\text{Time novel}-\text{Time familiar}}{\text{Time novel}+\text{Time familiar}}$$

#### Passive avoidance

The step-through passive avoidance test was performed according to the protocol of Gimenez De Bejar et al^25^., with slight modifications. The apparatus consisted of two chambers (dark and clear, 20 × 20 × 30 cm) separated by a guillotine door, with stainless steel rod floors (2 mm diameter, 0.5 cm apart) and illuminated by a 50 W bulb one meter above. Mice (n = 35) underwent two trials: a training trial and a test trial 24 h later. During training, mice were placed in the clear chamber, and upon entering the dark chamber, the door closed and a foot shock (1 mA, 5 s) was delivered. LLM (5, 10, or 20 mg/kg,i.p.) was administered 30 min prior to training, and scopolamine (3 mg/kg; i.p.) was given 30 min later to induce memory impairment. In a separate group, LLM was given alone 30 min prior to training with foot shock (1 mA, 5 s) to observe potential memory-enhancing effects without ceiling interference. During the test trial, latency to enter the dark compartment was measured (cut-off: 300 s).

#### Morris water maze

A spatial memory test was performed as previously described, with minor modifications^26,27^. Mice were put in a water-filled circular basin. The water was made opaque by using liquid peak milk. The water’s temperature was maintained at room temperature. The escape pulpit, which had a diameter of 25 cm, was positioned in the middle of the North-East quadrant. One centimeter was beneath the water’s surface. Throughout the learning trials, the animal remained atop the platform. To facilitate orienting, mice were placed on the platform 20 s before the first training session on the first training day. Following orientation, the animals were submerged in the liquid with their backs to the wall of the South-West area of the basin, and they were left to search for and locate the platform. When a mouse climbed onto the apparatus or sixty seconds had passed, the experiment was over. The mouse was taken out of the water right away if it found the pulpit before the 60 s were up. The mouse was gently led to the podium if it could not be found after 60 s of swimming. After being taken out of the water and patted dry with a hand towel, the mice were placed in a cage. To make sure the mice were completely dry, they were visually inspected. Every day, mice were subjected to two trials, with a 30-min break between each animal. Mice were given a probe trial session one day following the last training trial sessions. In this session, the platform was taken out of the pool, and the mice were given 120 s to swim in search of it. The swimming time in the pool quadrant where the platform had been previously installed was recorded. As a positive control, LLM (5, 10, and 20 mg/kg,i.p.) or DNP (3 mg/kg; p.o.) was administered one hour prior to the first trial session on each day that followed. SCP (3 mg/kg; i.p.) was administered to mice 30 min after LLM therapy to cause memory impairment. Only a solution of 0.02% (DMSO) was given to the control group.

### Statistical analysis

An analysis of the data was performed using R and Python Statistics, presenting the mean ± standard error of mean. Statistical differences across groups were assessed using one-way analysis of variance (ANOVA) followed by Tukey’s test. We also conduct *Bliss* synergy analysis to determine the synergistic effects. Statistical significance was defined as *p* values less than 0.05 or 0.01.

### In silico study

#### Selection and preparation of receptors

In this study, acetylcholinesterase (AChE) was chosen due to its known association with Alzheimer’s disease as reported in previous research^28^. The three-dimensional (3D) structure of AChE (PDB ID: 7E3H), specifically the A chain, was obtained from the RCSB Protein Data Bank an internationally recognized repository for 3D structural data of proteins and protein complexes^29^. The retrieved macromolecule underwent processing in PyMOL version 1.7.4.5 to remove unnecessary amino acid fragments and water. To minimize energy and optimize macromolecule, the Swiss-PDB Viewer software apply the GROMOS96 43B1 force field. Protein 3D coordinates have been isolated by removing water molecules, ligands, cofactors and ions, the non-protein components. The polar hydrogens were added Kollman charges assigned and the nonpolar hydrogens merged. The purified macromolecule saved in PDBQT format enabled molecular docking studies using AutoDock Vina.

#### Preparation of ligands

**Fig. 2:**
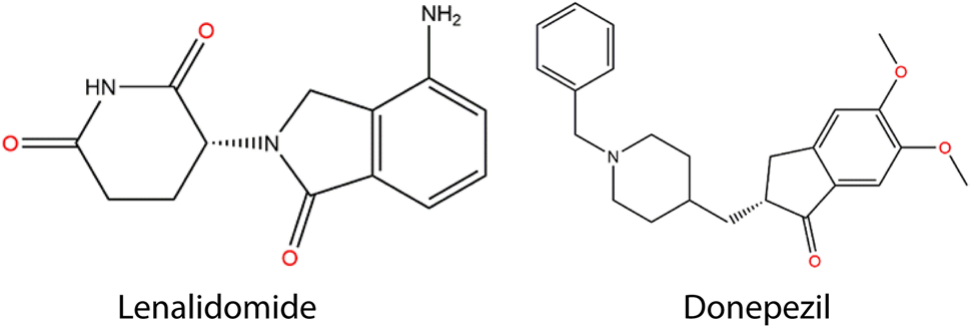
The chemical structures of lenalidomide and donepezil.

The 3D structures of LLM (PubChem ID: 216,326) and DNP (PubChem ID: 3152) were retrieved from the PubChem database in SDF format. Following retrieval, Chem3D Pro 21.0 was utilized to perform energy minimization of both ligands using Allinger’s MM2 force field method. The corresponding 2D structures of LLM and DNP are illustrated in Fig. 2.

#### Molecular docking

Molecular docking serves to evaluate the pharmacodynamic properties of drug candidates by assessing their binding affinity and molecular interactions, utilizing PyRx v0.8 software (Harini et al., 2024). The docking scores reflect the strength of interaction between the ligand and the target macromolecule. Additionally, the docking results help identify the specific binding sites of ligands on the receptor proteins (Gohlke and Klebe, 2002). In this in silico study, site-specific docking was employed. To accelerate the docking process, the dimensions of the grid box along the X, Y, and Z axes were − 43.368, 37.728, and − 30.313, respectively, to target the active or catalytic site, resulting in a computational speed increase of approximately 2000 fold. The ligand–protein complex was first saved in PDB format, which was then converted into PDBQT format for docking. PyRx v0.8 provided the docking scores as negative values, indicating the binding affinity. To visualize the ligand-receptor interactions and analyze non-bonded contacts, BIOVIA Discovery Studio v21.1.0 was employed.

#### Molecular dynamics

Molecular dynamics (MD) simulations were carried out using GROMACS version 2025.1 with default settings. Prior to simulation, all protein–ligand complexes were prepared by removing heteroatoms and resolving missing atoms or residues. The CHARMM36 all-atom force field was used for proteins, and ligand topologies were generated with CGenFF (https://app.cgenff.com/). Long-range electrostatics were calculated using the Particle Mesh Ewald (PME) method. A short-range Coulomb cutoff of 1.2 nm was applied, consistent with the CHARMM36 force field recommendations. PME settings included a Fourier grid spacing of 0.16 nm and cubic interpolation (PME order = 4). Electrostatics beyond the cutoff distance were treated via the full Ewald summation implemented in PME. Systems were placed in a cubic box with ≥ 1.5 nm padding and solvated with the CHARMM-modified TIP3P water model. Counterions (Na⁺/Cl⁻) were added to neutralize charge. Energy minimization used steepest descent (max 50,000 steps) with the Verlet cutoff scheme. Equilibration was performed with position restraints in NVT and NPT ensembles (100 ps each). Temperature was maintained at 300 K using the velocity-rescale thermostat; pressure was at 1 bar with the Parrinello-Rahman barostat. Long-range electrostatics were treated with PME, and Lennard–Jones (dispersion) interactions employed LJ-PME with a potential-shift modifier (no analytic dispersion correction). Short-range Coulomb and LJ cutoffs were 1.0–1.2 nm as indicated in the.mdp files below. All bonds to hydrogens were constrained with LINCS. Production runs of 100 ns used a 2 fs time step with the leap-frog integrator; coordinates and energies were saved every 10 ps. Post-simulation analyses (RMSD, RMSF, SASA, H-bonds, and Rg) were carried out with GROMACS tools and Python scripts.

#### Binding free energy calculations

##### Molecular mechanics Poisson-Boltzmann surface area (MM-PBSA) analysis

The binding free energies of the protein–ligand complex were calculated using the gmx_MMPBSA package (v1.6.4) (Valdés-Tresanco et al., 2021) (https://valdes-tresanco-ms.github.io/gmx_MMPBSA/), in conjunction with the GROMACS 2025.1 molecular dynamics framework. The CHARMM36 all-atom force field parameters were used, consistent with those applied in the MD simulations. A 100 ns production trajectory was used as the input for MM-PBSA calculations. The centered and post-processed trajectory (md_0_10_center.xtc) was analyzed from frame 1 to frame 10,000, with one frame extracted every 10 frames, resulting in a total of 1,000 evenly spaced frames covering the entire 100 ns simulation for binding free energy analysis. The Poisson-Boltzmann implicit solvent model (inp = 1) was applied for polar solvation free energy calculations, with dielectric constants of 2 for the solute and 80 for the solvent, and an ionic strength of 0.15 M. The non-polar solvation contribution was estimated using the solvent-accessible surface area (SASA) method (sasopt = 0). The van der Waals (VDWAALS) and electrostatic (EEL) terms were computed using the same cut-off distances and PME electrostatics parameters as in the MD production runs. Per-residue free energy decomposition (idecomp = 1) was performed to identify the contributions of individual residues to ligand binding. The gmx_MMPBSA_ana module was used for post-processing and visualization, providing energy breakdowns into van der Waals, electrostatic, polar solvation, and non-polar solvation components, as well as total binding free energies. All reported values are expressed as mean ± standard deviation over the analyzed frames.

#### Prediction of pharmacokinetics and drug-likeness

Pharmacokinetic (PK) properties play a vital role in evaluating and predicting the physiological effects of a compound, including its therapeutic benefits or potential adverse effects on specific biological processes. To assess the ADMET (Absorption, Distribution, Metabolism, Excretion, and Toxicity) profiles and drug-likeness of the compounds, the SwissADME online tool was employed.

#### Toxicity prediction

Toxicity prediction is significant in drug discovery as it helps in the recognition of compounds which are likely to be safe and effective to use. It also substantially reduces the risk of costly failures in the subsequent phases of drug development (Banerjee et al., 2024). The ProTox 3.0 web server provides a reliable prediction of various toxicity parameters for individual drug candidates. To execute the prediction, the canonical SMILES of the compounds are needed which can be obtained from the PubChem database.

## Results

### In vivo behavioral assessments

#### Y-maze

**Fig. 3:**
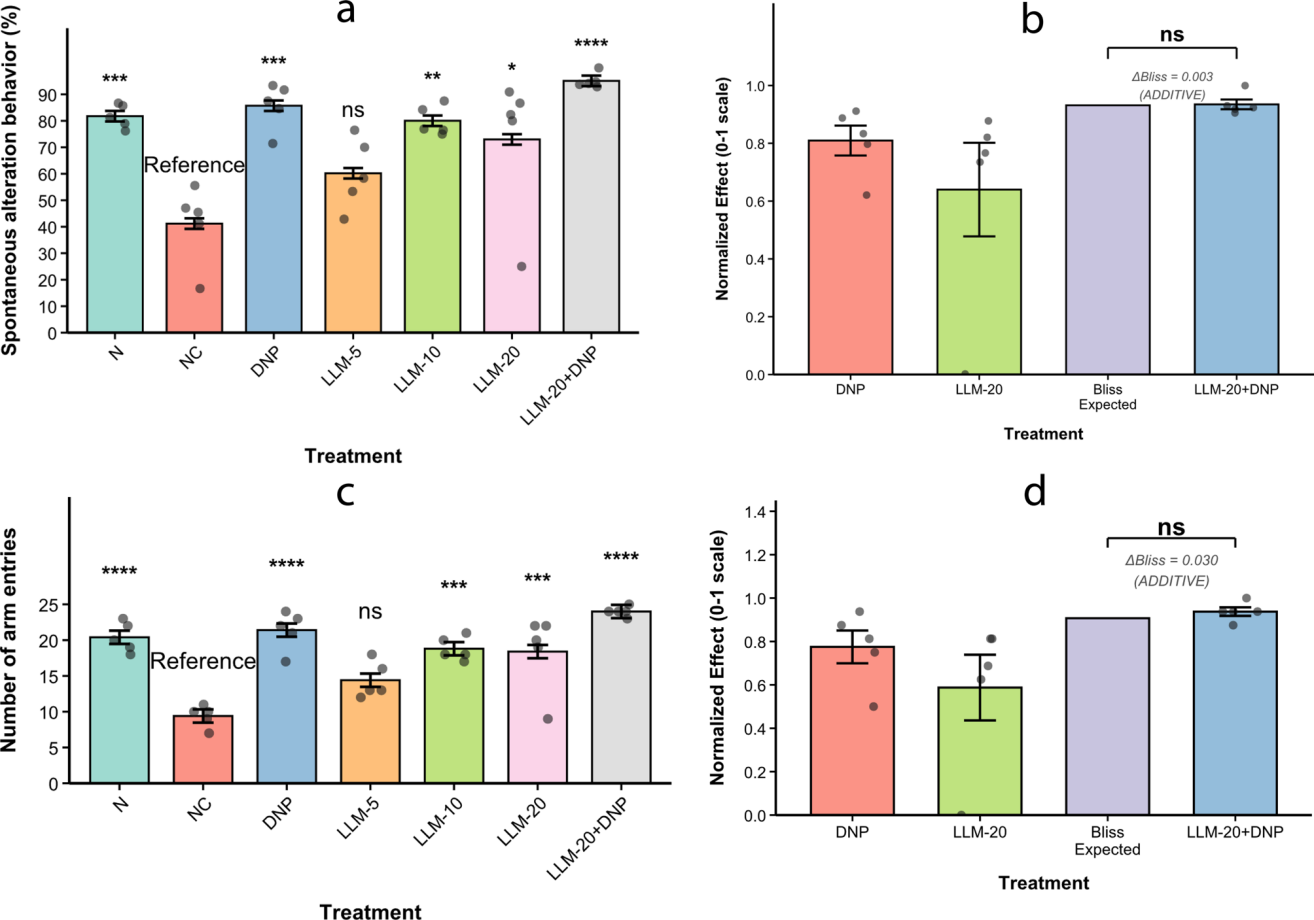
Effect of LLM on spontaneous alternation behavior in the Y‑maze test. (**a**) Percentage of spontaneous alternation in different treatment groups, (**b**) Bliss synergy analysis of alternation behavior, (**c**) Number of arm entries, (**d**) Bliss synergy analysis of Number of arm entries. Data are presented as mean ± SEM (n = 5). *p < 0.05, **p < 0.01, ***p < 0.001, ****p < 0.0001 vs. NC group. [N: Normal; NC: Negative control; DNP: Donepezil; LLM: Lenalidomide].

The Y-maze test was used to spontaneously assess spatial working memory through the alternation. Mice treated with SCP (the NC group) exhibited a significant decrease in the alternation percentage, 41.18 ± 13.12%, which is significantly lower than the untreated N group (N: 81.75 ± 3.97%, p < 0.05) confirming the impairment of spatial working memory. According to the positive control, treatment with DNP significantly improved alternation performance (85.71 ± 7.77%, p < 0.05 vs. NC). Higher doses of LLM resulted in better alternation behaviour. Mice given LLM at 5 mg/kg had a slight improvement of 60.20 ± 11.93% (p > 0.05 vs. NC). On the other hand, LLM at 10 mg/kg improved performance significantly to 80.02 ± 4.92% (p < 0.05 vs. NC), which was similar to the N group. The average of LLM at (20 mg/kg) was, however, slightly lower (72.99 ± 24.28%). Furthermore, inter-individual differences were greater. The highest level of alternation was shown by the group that received DNP (LLM-20 + DNP) in the presence of LLM (20 mg/kg), which gave 95.09 ± 2.53% that is significantly greater than other groups DNP group (p < 0.05) indicating a strong synergism. The results show that LLM ameliorates scopolamine-produced memory deficits in a dose-dependent manner and combination with DNP causes a significant improvement in spatial working memory performance (Fig. 3a). Results from Bliss Independence Model investigations showed that for the LLM-20 + DNP combination, the normalized effect was 0.936 which was not significantly different from the Bliss expected additive value 0.934 (ΔBliss = 0.003, a trivial 0.3% less than additive). The finding of synergy score of 0.020 with a non-significant one-sample t-test (t(3) = 0.191, p = 0.8580) suggests that LLM-20 and DNP had an additive interaction. Thus ruling out any synergistic or antagonistic effects. According to one-way ANOVA, the treatment groups did not significantly differ (F (3,12) = 3.25, p = 0.1476) further indicating this was purely additive effects (Fig. 3b).

The effect of the treatments on the arm entries were very significant as per one-way ANOVA (F(6,28) = 15.60, p < 0.000, η^2^ = 0.770. The (NC) group had significantly lower arm entries (9.6 ± 0.4) than the reference (N) group (20.6 ± 0.7) (****p < 0.0001, indicating cognitive impairment was successfully induced. The DNP treatment alone (21.4 ± 0.7) and LLM-20 + DNP (24.0 ± 0.7) did not significantly differ from the N (ns), showing preserved cognitive function. Notably, post-hoc Tukey’s test confirmed that NC was significantly different from all other treatment groups except LLM-5 (p < 0.0001 for most comparisons), while LLM-5, LLM-10, and LLM-20 groups showed intermediate performance with significantly fewer arm entries than the N (LLM-5: p = 0.00625; LLM-10: p = 0.00016; LLM-20: p = 0.0003). The LLM-20 + DNP combination showed superior performance compared to LLM-20 alone (p = 0.04417), though it did not significantly differ from LLM-10 (p = 0.07355) (Fig. 3c). The Bliss Independence Model analysis revealed an observed normalized effect of 0.938 for the LLM-20 + DNP combination, which did not significantly differ from the Bliss expected value (ΔBliss = 0.030, representing 3.3% deviation), with a synergy score of 0.020 indicating an additive rather than synergistic effect (ns, one-sample t-test: t(3) = 1.554, p = 0.1969) (Fig. 3d).

#### Novel object recognition

**Fig. 4:**
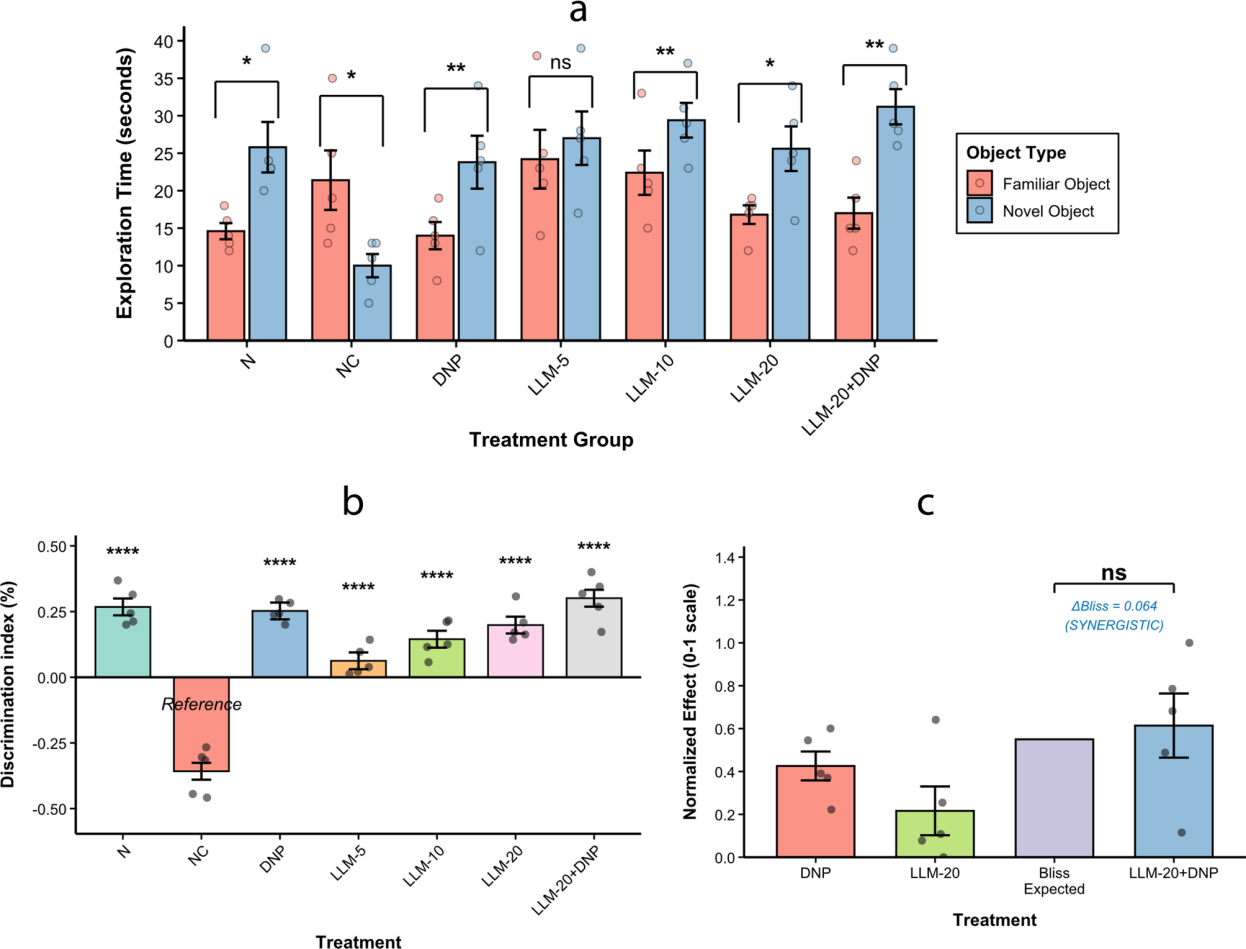
Novel object recognition test performance. (**a**) Exploration time (seconds) spent with the familiar and novel objects, (**b**) Discrimination index calculated as (Time‑novel − Time‑familiar)/(Time‑novel + Time‑familiar), (**c**) Bliss synergy analysis of novel object recognition test performance. Data are mean ± SEM (n = 5). *p < 0.05, **p < 0.01, ***p < 0.001, ****p < 0.0001 vs. NC group.[N: Normal; NC: Negative controll; DNP: Donepezil; LLM: Lenalidomide].

To assess recognition memory, mice underwent the novel object recognition test where exploration time and discrimination indices were measured (Fig. 4a). Two-way ANOVA revealed significant main effects of treatment (F = 3.64, p = 0.004), object type (F = 16.52, p < 0.001), and a significant treatment × object type interaction (F = 4.62, p = 0.0007). (N) exhibited intact recognition memory with a discrimination index of 0.283 ± 0.026 (mean ± SEM), significantly different from the reference baseline (p < 0.0001). In contrast, the negative control group (NC) showed severely impaired recognition memory with a negative discrimination index of − 0.342 ± 0.054, indicating a reversed preference pattern and representing the worst performance among all groups (NC vs all treatments: p < 0.0001). One-way ANOVA on discrimination indices confirmed significant treatment effects (F(6,28) = 53.82, p < 0.001, η^2^ = 0.920). Post hoc Tukey’s tests revealed that DNP treatment (DI = 0.198 ± 0.019) partially restored recognition memory but remained significantly impaired compared to NC (p < 0.0001). LLM-5 treatment (DI = 0.088 ± 0.034) showed minimal improvement and was significantly worse than DNP (p = 0.003). Both LLM-10 (DI = 0.143 ± 0.028) and LLM-20 (DI = 0.197 ± 0.020) treatments demonstrated significant memory restoration (both p < 0.0001 vs NC), with LLM-20 performing comparably to DNP (p = 0.878). Notably, the combination treatment LLM-20 + DNP produced the most robust effect with a discrimination index of 0.246 ± 0.024 (p < 0.0001 vs NC), which was significantly superior to LLM-10 (p = 0.020) and LLM-5 (p < 0.001), and showed no significant difference from normal controls (p = 0.987) (Fig. 4b). Bliss independence analysis demonstrated a synergistic interaction between LLM-20 and DNP (F = 7.98, p = 0.014), with the observed effect (0.60 ± 0.18, normalized scale) exceeding the expected additive effect (0.54) by 11.6% (ΔBliss = 0.064), confirming that the combination therapy produces synergistic enhancement of recognition memory beyond simple additive effects (Fig. 4c).

#### Passive avoidance

**Fig. 5:**
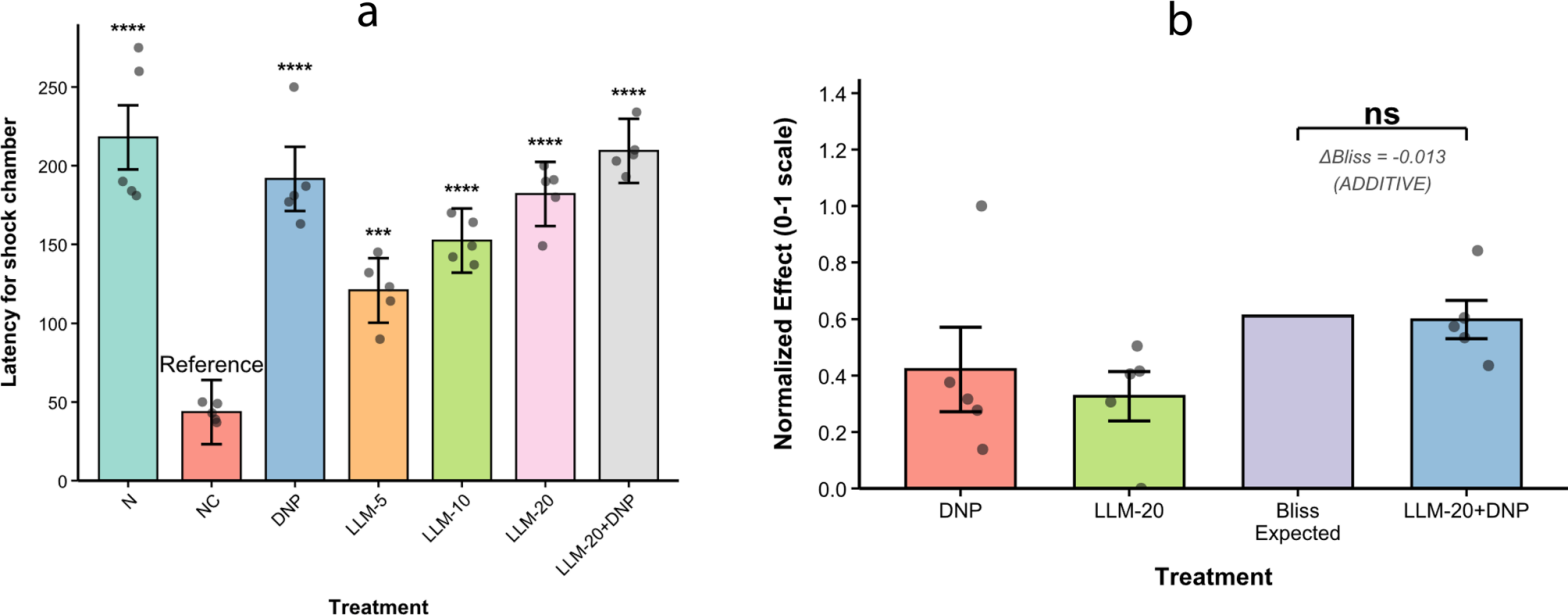
Passive avoidance test results. (**a**) Step‑through latency (seconds) during the retention trial, (**b**) Bliss synergy analysis of passive avoidance test results. Data are mean ± SEM (n = 5). *p < 0.05, **p < 0.01, ***p < 0.001, ****p < 0.0001 vs. NC group. [N: Normal; NC: Negative controll; DNP: Donepezil; LLM: Lenalidomide].

In the passive avoidance task, scopolamine-treated mice (NC group) displayed a significant reduction in step-through latency (43.6 ± 5.2 s) compared to the N untreated group (N: 218 ± 40.79 s), indicating cognitive impairment induced by scopolamine (p < 0.05). Mice pre-treated with DNP showed a significant improvement in latency (191.6 ± 30.25 s, p < 0.05 vs. NC), demonstrating the protective effect of the standard drug. Treatment with LLM resulted in a dose-dependent increase in latency to enter the dark chamber, indicating amelioration of memory impairment. Mice receiving LLM at 5 mg/kg (LLM-5) showed a moderate improvement (120.8 ± 18.50 s), while LLM at 10 mg/kg (LLM-10) and 20 mg/kg (LLM-20) showed statistically significant improvements (152.4 ± 12.66 and 182 ± 17.67 s, respectively; p < 0.05 vs. NC). The latency values of the highest dose (LLM-20) appeared similar to DNP standard group. In addition, the maximum effect (209.4 ± 13.57 s) was observed in mice treated with a combination of LLM (20 mg/kg) and DNP (LLM-20 + DNP), with latency significantly greater than all the other groups (p < 0.05 vs. NC, DNP and LLM-5). There is a possibility that LLM and DNP have a synergistic effect on memory restoration. Thus, the results suggest that LLM attenuates cognitive deficits in scopolamine-induced mice and its efficacy increases in a dose-dependent manner (Fig. 5a) and it has positive synergistic effects with DNP.

LLM-20 + DNP exhibited no synergistic effect (ns, ΔBliss = − 0.013, representing only 2.1% drift from expected additive effect). The combined effect that was observed (0.598) was similar to the Bliss expected value (0.611) indicating purely additive interaction. The results of the analysis showed no difference between the treatments (one-way ANOVA: F(2,12) = 1.64, p = 0.2335; one-sample t-test: p = 0.8595), demonstrating that the LLM and DNP acts independently without synergy or antagonism (Fig. 5b).

#### Morris water maze

**Fig. 6:**
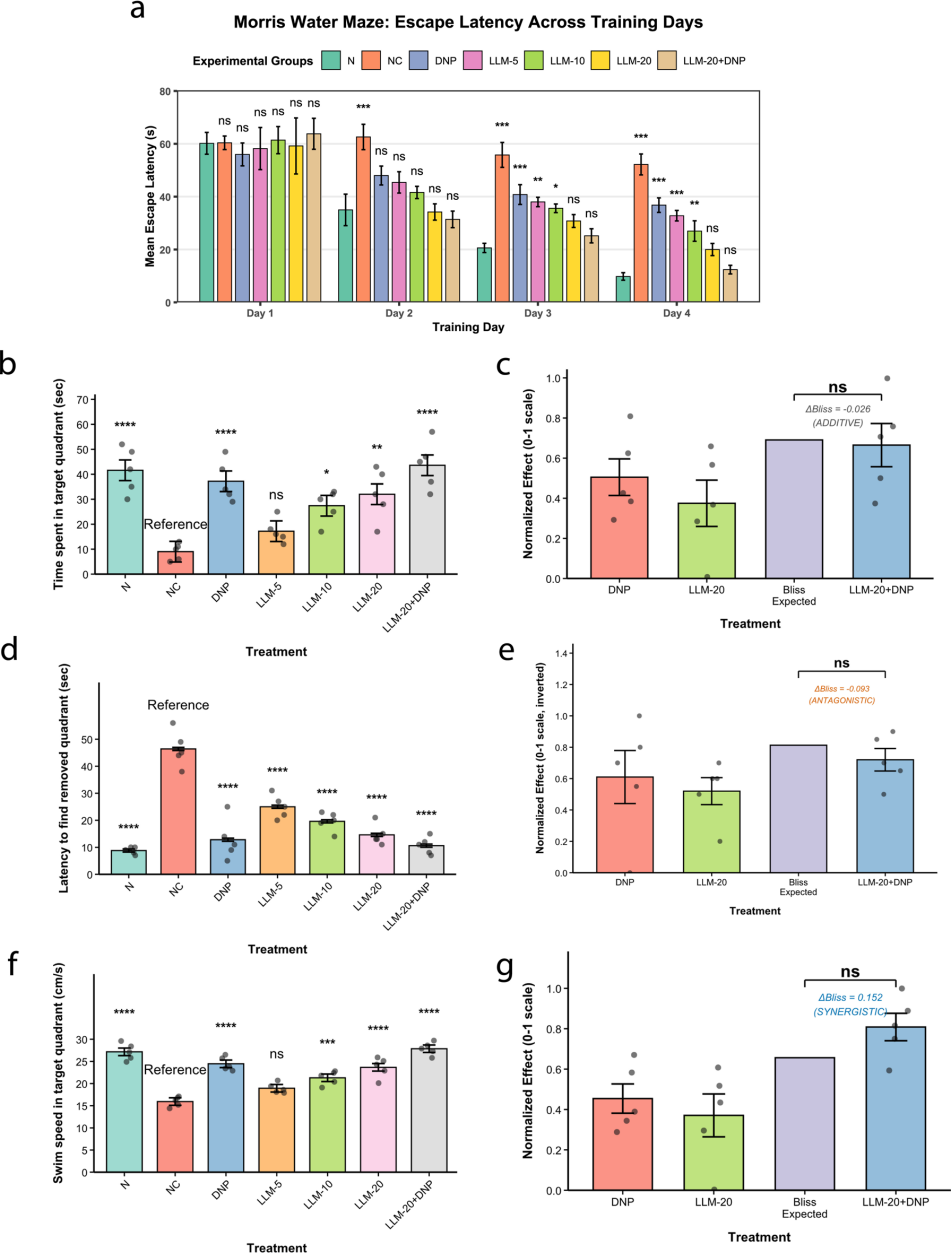
Morris water maze performance. (**a**) Escape latency across four consecutive training days, (**b**) Time spent in target quadrant, (**c**) Bliss synergy analysis of time spent in the target quadrant, (**d**) Latency to find removed quadrant, (**e**) Bliss synergy analysis of latency to find removed quadrant, (**f**) swimming speedin target quadrant, and (**g**) Bliss synergy analysis of swimming speedin target quadrant. Data are mean ± SEM (n = 5). *p < 0.05, **p < 0.01, ***p < 0.001, ****p < 0.0001 vs. NC group. [N: Normal; NC: Negative controll; DNP: Donepezil; LLM: Lenalidomide; NS: Non significance].

In Morris water maze test, the scopolamine-induced spatial memory deficit in the (N) group was confirmed by significantly higher latency to reach the removed platform (46.4 ± 5.95 s) and significantly reduced time spent in the target quadrant (9.0 ± 3.03 s), thereby confirming impaired spatial learning and memory. On the other side, DNP standard treatment significantly improved performance by decreasing latency to 191.6 ± 30.25 s (p < 0.01 vs NC) and by increasing quadrant time to 37.2 ± 7.30 s (p < 0.01 vs NC). LLM treatment alone showed cognitive recovery in a dose-dependent manner. LLM at 5 mg/kg reduced latency to 25.0 ± 3.85 s and increased target quadrant time to 17.2 ± 4.35 s (p < 0.05 vs. NC). LLM at 10 mg/kg led to further improvements (latency: 19.6 ± 3.13 s; quadrant time: 27.4 ± 5.89 s; *p* < *0.01* vs. NC), while LLM-20 showed even stronger effects (latency: 14.6 ± 3.44 s; quadrant time: 32.0 ± 9.23 s; p < 0.01 vs. NC), nearing the performance of DNP. The combination therapy (LLM-20 + DNP) yielded the best outcome, with latency reduced to 10.6 ± 2.87 s and quadrant time increased to 43.6 ± 8.62 s (p < 0.01 vs. NC), closely resembling the (N) untreated group (latency: 8.8 ± 1.17 s; quadrant time: 41.6 ± 8.26 s) (Fig. 6b,d). Furthermore, data from four consecutive training days (Fig. 6a) indicated a consistent decline in escape latency across sessions for all treatment groups, particularly in LLM-10, LLM-20, and LLM-20 + DNP, reflecting enhanced learning ability (*p* < *0.05 to p* < *0.01*).

The findings of the time spent in the target quadrant showed no significant derived synergy effect due to combination LLM-20 + DNP (ns, ΔBliss = − 0.026, p = 0.1914). The combined effect observed (0.665) corresponded closely to the Bliss expected (0.691), suggesting an additive interaction rather than synergistic one. According to one-way ANOVA, no significant difference was found between treatments (F(2,12) = 1.00, p = 0.1914) (Fig. 6c). Also, the inverted scale analysis for latency to find removed quadrant, revealed no synergy, with an antagonistic trend of the combination (ΔBliss = − 0.093, p = 0.6615). The observed effect was 0.720, whereas the Bliss expected value was 0.813, although this difference was not statistically significant. No significant treatment differences were detected with a one-way ANOVA analysis (F(2,12) = 0.73, p = 0.6615). Thus, it appears the two compounds act independently without any meaningful synergism as shown in (Fig. 6e).

In the Morris water maze experiment, swim velocity was analyzed to rule out motor confounds in cognitive improvements seeing that the two are correlated. The swimming speed (15.94 ± 0.46 cm/s) of scopolamine-treated (NC) animals was significantly reduced compared to (N) animals (27.16 ± 0.84 cm/s, p < 0.001). According to the study’s findings, treatment with DNP raised the swim speed to almost normal at 24.46 ± 0.71 cm/s (p < 0.001 vs. NC). The treatment with LLM resulted in a notable improvement in the speed of the swimming motion in a dose-dependent manner showing values of LLM-5 (18.94 ± 0.48 cm/s, p > 0.05 vs. NC), LLM-10 (21.30 ± 0.64 cm/s, p < 0.001 vs. NC), and LLM-20 (23.66 ± 1.04 cm/s, p < 0.001 vs. NC). According to the data, the combination treatment (LLM-20 + DNP) gave the highest speed of swimming 27.86 ± 0.68 cm/s, which was not significantly different from the N group (p = 0.992). Statistical analysis by one-way ANOVA revealed a significant effect of treatment on the outcome (F(6,28) = 35.88, p < 1 × 10⁻^1^⁰). The findings indicate that the cognitive improvements noted in the treatment groups were not due to changes in motor performance, supporting the neuroprotective and cognitive enhancing potential of LLM alone and in combination with DNP (Fig. 6f).

The combination of LLM-20 + DNP demonstrated a synergistic trend (ΔBliss = 0.152, representing 23.1% deviation from expected additive effect), though this did not reach statistical significance (ns, p = 0.0072 by one-sample t-test but p = 0.6890 by one-way ANOVA). The observed combined effect (0.808) exceeded the Bliss expected value (0.657), suggesting potential synergy. However, the non-significant result from ANOVA (F(2,12) = 7.86, p = 0.0072) indicates this synergistic interaction was not robustly established at the statistical threshold used (Fig. 6g).

### In silico findings

#### Molecular docking

In this study, molecular docking was conducted to investigate the binding affinity and interaction profiles of two ligands, DNP and LLM, with the target enzyme (AChE). The docking results provide insights into their potential as therapeutic agents targeting AChE, a key enzyme implicated in neurological conditions such as Alzheimer’s disease. The ligand DNP, a well-established acetylcholinesterase inhibitor currently available in the pharmaceutical market, showed a strong binding affinity with a docking score of − 11.5 kcal/mol. It formed critical hydrogen bonds with residues PHE A:295 (2.06 Å) and ARG A:296 (2.86 Å), which are important for ligand stability within the active site. Additionally, DNP exhibited multiple π interactions, including π-sigma and π-π stacking with residues TYR A:341, TRP A:86, and TRP A:286, alongside alkyl and π-alkyl interactions with LEU A:289, TYR A:337, and PHE A:338. These interactions confirm DNP’s strong and specific binding, validating its efficacy as a standard AChE inhibitor. On the other hand, the ligand LLM, which is currently under investigation, displayed a moderate binding affinity with a docking score of − 8.7 kcal/mol. LLM formed three hydrogen bonds with TYR A:133 (1.82 Å), TYR A:337 (2.86 Å), and GLY A:126 (3.60 Å), as well as a π-π T-shaped interaction with TYR A:337. While its binding energy was less favorable than DNP, LLM demonstrated significant interactions with key residues in the active site, suggesting a promising binding profile. The results indicate that LLM has the potential to serve as a novel AChE inhibitor, warranting further in vitro and in vivo studies to explore its efficacy and safety. Its ability to interact effectively with AChE suggests it could be developed into a future therapeutic agent for neurodegenerative diseases, possibly offering alternative or complementary benefits to existing drugs like DNP (Table 1andFig. 7).

**Table 1:** Molecular docking results of donepezil and lenalidomide with acetylcholinesterase, showing docking scores (kcal/mol) and key interacting residues.

Macromolecule	Ligand	Docking score (kcal/mol)	Amino acid (AA) residues	
			Hydrogen bond with length	Other interacted bonds
AChE	DNP	− 11.5	PHE A:295 (2.05958) ARG A:296 (2.85957)	TYR A:341(Pi-Sigma) TRP A:86 (Pi-Pi Stacked) TRP A:286 (Pi-Pi Stacked) LEU A:289 (Alkyl) TRP A:286 (Pi-Alkyl) TYR A:337 (Pi-Alkyl) PHE A:338 (Pi-Alkyl)
	LLM	− 8.7	TYR A:133 (1.82416) TYR A:337 (2.8566) GLY A:126 (3.6042)	TYR A:337 (Pi-Pi T-shaped)

*LLM* Lenalidomide, *DNP* Donepezil, *AChE* Acetylcholinesterase.

**Fig. 7:**
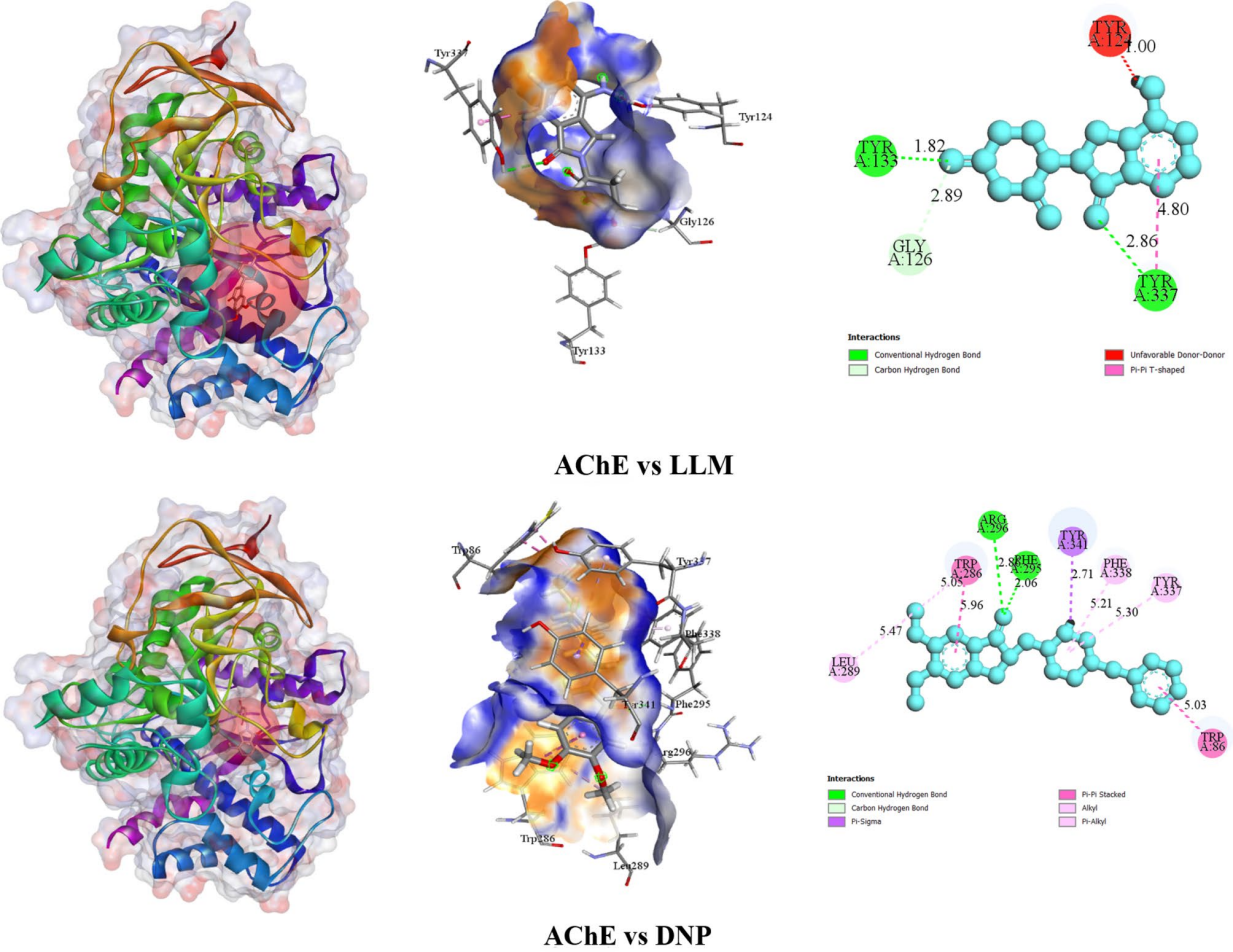
Structures of molecular docking donepezil and lenalidomide with acetylcholinesterase receptor in two and three dimensions (2D and 3D). [LLM: Lenalidomide; DNP: Donepezil; AChE: Acetylcholinesterase].

#### Molecular dynamics results

To validate the stability of the ligand–protein complexes identified through molecular docking and to gain insights into their dynamic behavior at an atomic level, we performed 100 ns molecular dynamics (MD) simulations for the AChE-LLM (Test compound) complex, the AChE-DNP (Standard) complex, and the Apo-AChE (receptor alone) system.

##### Root mean square deviation (RMSD)

**Fig. 8:**
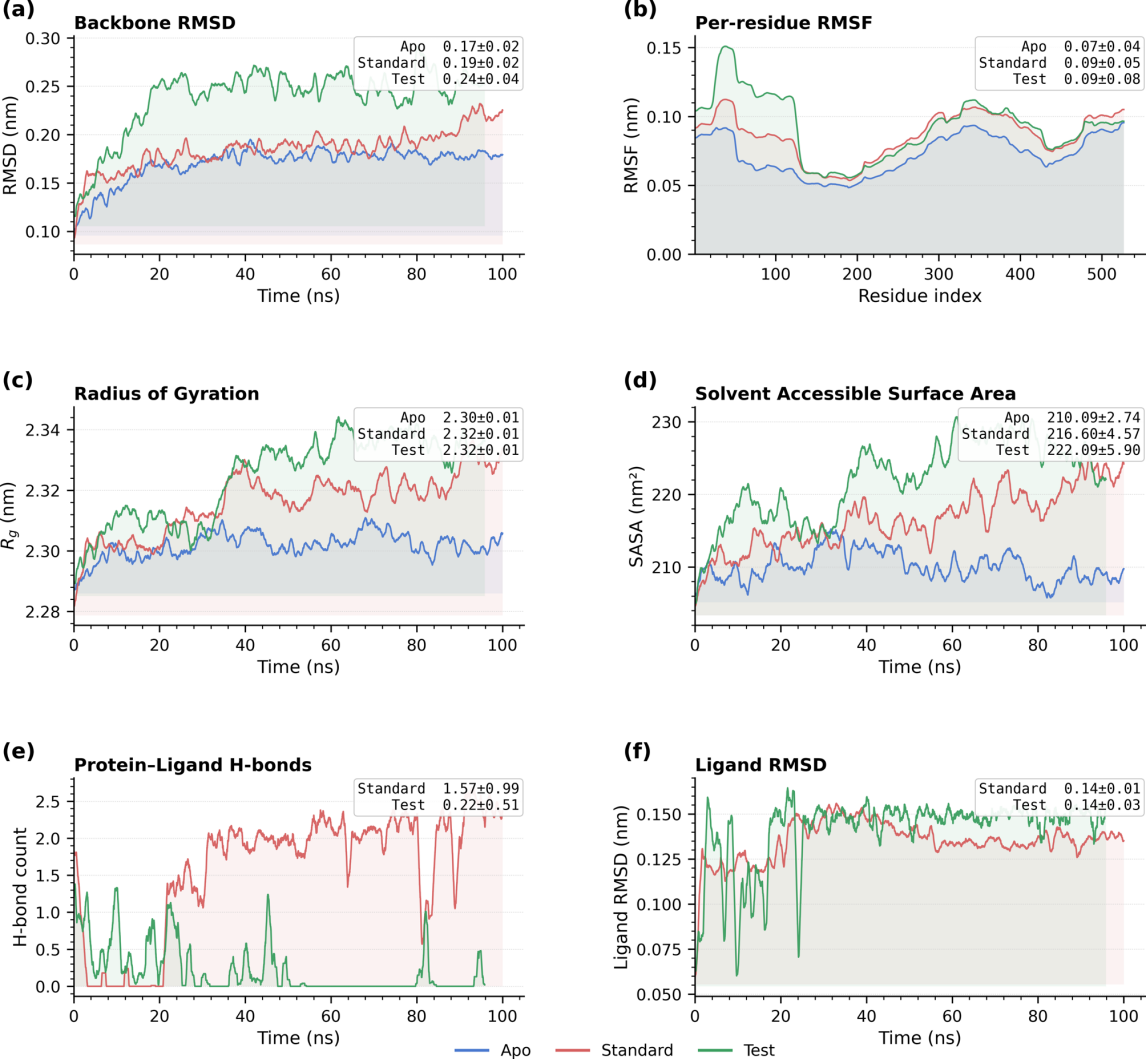
Molecular dynamics simulation profiles of the Apo-AChE, AChE-DNP (standard), and AChE–LLM (test compound) complexes over 100 ns. (**a**) RMSD of the protein backbone, (**b**) RMSF of individual residues, (**c**) (Rg) reflecting overall compactness, (**d**) Solvent-accessible surface area, (**e**) Number of hydrogen bonds between the protein and ligands (DNP or LLM) over time, (**f**) Time‑dependent hydrogen bond occupancy profile highlighting persistent interactions in the AChE-LLM complex. [RMSD: Root-mean-square deviation; RMSF: Root-mean-square fluctuation; Rg: Radius of gyration; LLM: Lenalidomide; DNP: Donepezil; AChE: Acetylcholinesterase].

RMSD curves in molecular dynamics simulations represented the fluctuations in protein conformation, reflecting the movement of the receptor–ligand complexes. A higher RMSD value indicated greater instability and pronounced fluctuations, while a lower value indicated a more stable complex. The RMSD of the protein backbone atoms was calculated to assess the overall structural stability and conformational changes of the complexes throughout the simulation trajectory (Fig. 8a). All three systems Apo-AChE, AChE-Standard, and AChE-Test compound, exhibited an initial rise in RMSD during the first ~ 10–20 ns, which is typical as the system equilibrates from its initial coordinates. Following this period, the Apo-AChE and the AChE-Standard complexes demonstrated remarkable stability, with their RMSD values plateauing at approximately ~ 0.25 and ~ 0.30 nm, respectively, for the remainder of the simulation. These low and stable RMSD values indicate minimal backbone deviation and high complex stability. The AChE-Test compound complex exhibited a slightly higher average RMSD of ~ 0.35 nm and showed greater fluctuations, particularly between 40–60 ns, before stabilizing. This suggests that while the LLM complex is stable, it induces slightly more flexibility in the protein structure compared to the tightly bound DNP complex.

##### Root mean square fluctuation (RMSF)

The RMSF was analyzed to evaluate the flexibility of individual amino acid residues (Fig. 8b). The RMSF profiles for all three systems were largely superimposable, indicating that ligand binding did not induce major changes in the local flexibility of the protein. Peaks of higher fluctuation were observed in the loop regions (e.g., residues 70–80, 120–140), which is expected due to the inherent flexibility of these solvent-exposed areas. Crucially, the residues comprising the active site (e.g., TRP86, PHE295, TYR337, HIS447) showed consistently low fluctuations across all systems. This confirms that the binding of both DNP and lenalidomide does not destabilize the crucial catalytic architecture of the AChE enzyme.

##### Radius of gyration (Rg)

The Rg, which measures the compactness of the protein structure, remained consistent throughout the simulation for all systems (Fig. 8c). The Apo-AChE and the AChE-Standard complex maintained nearly identical Rg values around ~ 2.15 nm, indicating a highly compact and stable fold. The AChE-Test compound complex showed a marginally higher Rg value (~ 2.18 nm), which correlates with its higher RMSD and suggests a minor loosening of the overall protein structure upon lenalidomide binding. However, the absence of any significant upward drift in Rg confirms that no large-scale unfolding or loss of structural integrity occurred in any of the systems.

##### Solvent-accessible surface area (SASA)

The SASA was monitored to track changes in the protein’s solvent exposure (Fig. 8d). The SASA values for the Apo and Standard complexes were stable and virtually overlapping throughout the simulation. The Test compound complex displayed a slightly higher SASA, which is consistent with its higher Rg and RMSD values. This indicates that lenalidomide binding might expose a slightly larger surface area to the solvent, potentially due to its distinct binding mode compared to DNP.

##### Hydrogen bond analysis

The number of hydrogen bonds formed between the protein and the ligands is a key metric for assessing binding stability (Fig. 8e). The AChE-Standard (DNP) complex maintained a consistently higher average number of hydrogen bonds (averaging between 2–4 throughout the trajectory), corroborating the strong binding affinity observed in the docking studies. The AChE-Test compound (LLM) complex formed fewer but stable hydrogen bonds (averaging 1–2), which were sustained for significant portions of the simulation. This persistent hydrogen bonding, particularly with key residues like TYR337, is critical for stabilizing the complex and explains the moderate but significant binding affinity of LLM.

##### Ligand RMSD 

The ligand RMSD analysis over 100 ns shows that both the standard and test ligands remain stable within the binding pocket after initial equilibration. The standard ligand exhibits smooth and consistent RMSD values (~ 0.14–0.16 nm), indicating stable binding. The test ligand shows higher short-term fluctuations but remains within a comparable RMSD range (~ 0.12–0.17 nm) without any upward drift. Overall, the results indicate stable binding of the test ligand, comparable to the standard ligand (Fig. 8f).

##### Binding free energy

**Fig. 9:**
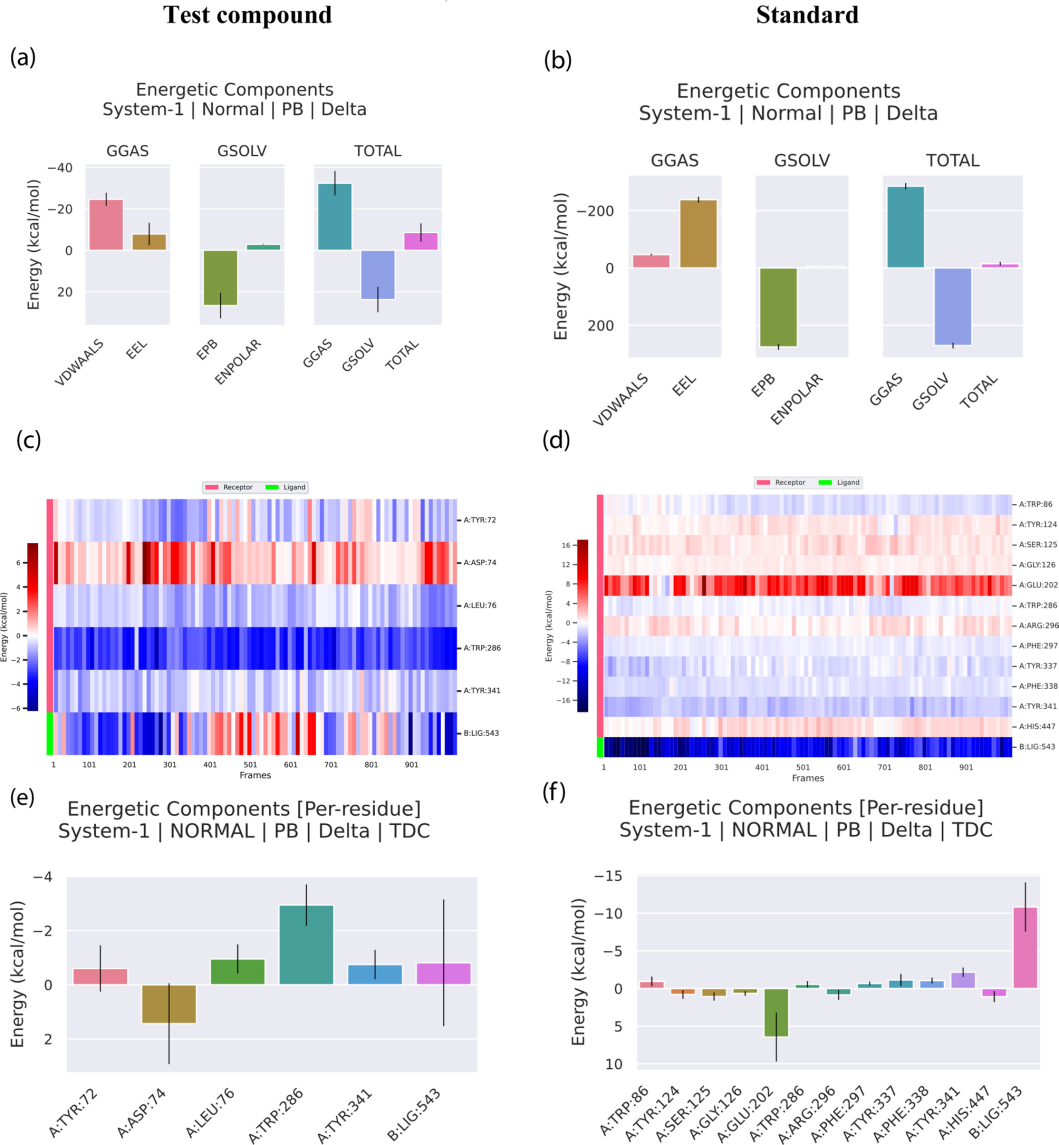
Molecular Mechanics/Poisson-Boltzmann Surface Area (MM/PBSA) binding free energy decomposition analysis for the test compound and the standard ligand in System-1. The total binding free energy (ΔG_bind) and its individual energetic components, including van der Waals (VDWAALS), electrostatic (EEL), polar solvation (EPB), and nonpolar solvation (ENPOLAR) energies, are compared between the two ligands. Per-residue energy contributions are illustrated using heat maps to identify key residues involved in ligand binding, and bar plots are provided for selected residues exhibiting significant energetic contributions.

The molecular mechanics Poisson-Boltzmann surface area (MM-PBSA) method was employed to calculate the binding free energy (ΔG_bind) of the complexes, providing a quantitative measure of binding affinity (Fig. 9a-f). The results confirmed that both ligands form stable complexes with AChE, with strongly negative ΔG_bind values indicating spontaneous binding. The AChE-Standard complex exhibited a binding free energy of (− 7.82) kcal/mol. The AChE-Test compound complex also showed a favorable, though less negative, binding free energy of (− 6.89) kcal/mol.

Energy decomposition analysis revealed that the binding for both complexes was primarily driven by van der Waals interactions (VDWAALS) and non-polar solvation energy (ENPOLAR), which are typical for hydrophobic binding pockets like that of AChE. The electrostatic component (EEL) also contributed favorably, while the polar solvation energy (EPB) term was unfavorable, as is common for charged or polar ligands desolvating upon binding. Per-residue decomposition analysis identified key residues (e.g., TYR337, TRP86, PHE295) that made the most significant energetic contributions to ligand binding, aligning perfectly with the interaction profiles identified in the molecular docking study.

#### Pharmacokinetics

The (PK) properties and drug-likeness of LLM was determined in silico using the SwissADME web tool and compared with the standard drug (DNP) to evaluate its potential as a CNS agent. The prediction of the two compounds was high gastrointestinal absorption, with zero violation of Lipinski’s Rule of Five, indicating good oral bioavailability. However, notable differences were noted which could affect their therapeutic profiles. The classification of two exploratory drugs was significantly different due to their structural differences. LLM, being smaller in size, and having lower molecular mass (259.2 g/mol) and higher polarity (TPSA = 92.50 Å^2^) was classified as ‘Very soluble’. On the contrary, the larger and more lipophilic DNP was classified as ‘Moderately soluble’. This is because it was 379.49 g/mol and TPSA = 38.7 Å^2^). Critically, LLM demonstrated a significantly lower predicted CNS permeability (log PS = − 3.074) compared to DNP (log PS = − 1.464). Furthermore, LLM presented a more favorable drug interaction profile, showing no inhibition of key cytochrome P450 enzymes (CYP1A2, CYP2C19, CYP2D6, CYP3A4), whereas DNP was predicted to inhibit CYP2D6 and CYP3A4. LLM was also not predicted to be a substrate for P-glycoprotein (P-gp) or renal OCT2, unlike DNP. These results suggest that while LLM has excellent solubility and a clean interaction profile, its penetration into the brain may be slightly low compared to the established CNS drug DNP. Table 2 represents all the pharmacokinetic properties of ligands.

**Table 2:** Comparison of pharmacokinetic properties of lenalidomide and donepezil estimated by SwissADME and pkCSM.

Parameters		Lenalidomide	Donepezil
Physicochemical properties	Molecular mass (g/mol)	259.2 g/mol	379.49 g/mol
	Number of heavy atoms	19	28
	Number of aromatic heavy atoms	6	12
	Number of rotatable bonds	1	6
	Number of H-bond acceptors	3	4
	Number of H-bond donors	2	0
	Molar Refractivity	74.93	115.31
	TPSA	92.50 Å^2^	38.7 Å^2^
Lipophilicity	Log Po/w (MLOGP)	0.54	3.06
	Log Po/w (iLOGP)	1.06	3.92
	Log Po/w (XLOGP)	− 0.50	4.28
Water Solubility	Solubility class	Very soluble	Moderately soluble
	Log s (ESOL)	− 1.30	− 4.81
Pharmacokinetics	GI absorption	High	High
	Caco2 permeability (log Papp in 10–6 cm/s)	0.138	1.273
	Intestinal absorption (human) (% Absorbed)	75.835	93.707
	VDss (human) (log L/kg)	0.114	1.266
	CNS permeability (log PS)	− 3.074	− 1.464
	Total Clearance (log ml/min/kg)	0.265	0.987
	Renal OCT2 substrate	No	Yes
	AMES toxicity	No	No
	Max. tolerated dose (human) (log mg/kg/day)	− 0.168	− 0.217
	BBB permeant	− 0.251	0.157
	P-gp substrate	No	Yes
	CYP1A2 inhibitor	No	No
	CYP2C19 inhibitor	No	No
	CYP2D6 inhibitor	No	Yes
	CYP4A4 inhibitor	No	Yes
Drug-likeness	Lipinski	Yes, 0 violations	Yes, 0 violations
	Bioavailability score	0.55	0.55

GI Gastrointestinal, VDss Volume of distribution at steady sate, CNS Central nervous system, BBB Blood–Brain Barrier, P-gp P-glycoprotein.

#### Toxicity

The in silico toxicological profiles of LLM and DNP were predicted using the ProTox 3.0 web server. Both compounds were predicted to fall under Toxicity Class 4 (LD₅₀ between 300–2000 mg/kg), with LLM exhibiting a higher predicted LD₅₀ value (700 mg/kg) than DNP (505 mg/kg), suggesting a potentially wider therapeutic window for LLM. A notable finding was that LLM was predicted to be inactive in several crucial toxicity endpoints where DNP was predicted to be active, including carcinogenicity, cytotoxicity, and immunotoxicity. Both molecules were predicted to be inactive in hepatotoxicity, mutagenicity, cardiotoxicity, and nephrotoxicity. Conversely, both were predicted to be active in other endpoints such as respiratory toxicity, neurotoxicity, and clinical toxicity. These predictions indicate that LLM may possess a more favorable preliminary toxicity profile than DNP, particularly regarding long-term risks like carcinogenicity and general cell toxicity. Table 3 shows all the toxicological properties of Ligands.

**Table:** Table 3. Comparison of toxicological properties of lenalidomide and donepezil estimated by ProTox 3.0.

	**Parameters**	**Report/predicted value**	
		**Lenalidomide**	**Donepezil**
Toxicity	Toxicity class	4	4
	LD_50_ (mg/kg)	700	505
	Carcinogenicity	Inactive	Active
	Cytotoxicity	Inactive	Active
	Immunotoxicity	Inactive	Active
	Hepatotoxicity	Inactive	Inactive
	Mutagenicity	Inactive	Inactive
	Respiratory toxicity	Active	Active
	Cardiotoxicity	Inactive	Inactive
	Nephrotoxicity	Inactive	Inactive
	Neurotoxicity	Active	Active
	BBB-barrier	Active	Active
	Ecotoxicity	Inactive	Active
	Clinical toxicity	Active	Active
	Nutritional toxicity	Inactive	Inactive

## Discussion

The gradual loss of cognitive function associated with amyloid-beta plaques, neurofibrillary tangles, cholinergic deficiency, and chronic neuroinflammation makes cholinergic dysfunction a significant worldwide health concern^30^^[,31^. The only available treatments are AChEIs and NMDA receptor antagonists, which provide symptomatic relief but frequently have ineffectiveness and side effects (Muñoz-Torrero D., 2008). In a scopolamine-induced mouse model of cholinergic dysfunction and cognitive impairment, this study offers strong combined evidence, both in silico and in vivo, that LLM, a thalidomide derivative with well-established immunomodulatory and anti-inflammatory qualities, improves cognitive deficits mainly by inhibiting AChE.

We selected the scopolamine-induced cognitive impairment model for several reasons. First, it is a well-validated, reproducible, and efficient paradigm for screening potential cognitive enhancers, particularly those targeting the cholinergic system, which remains central to symptomatic treatment in AD^32,33^. Scopolamine rapidly and reliably causes memory deficits through competitive blockade of muscarinic acetylcholine receptors, particularly the M1 subtype, thus disrupting essential cholinergic neurotransmission for learning, attention, and memory^33,34^. Apart from blocking receptors, SCP allegedly increases oxidative stress and neuroinflammatory processes, thus contributing to cognitive impairment^35,36^. As these features make the model very sensitive for the detection of compound able to restore cholinergic function, it is a useful tool for preliminary proof-of-concept screening of cognition enhancers^32^. Nonetheless, it continues to be a sharp, drug-induced model that fails to produce main pathological hallmarks of AD such as amyloid-β deposition, tau hyperphosphorylation or chronic neuroinflammation^37,38^. As a result, the marked improvements induced by lenalidomide suggest its substantial potential for reversing cholinergic dysfunction in this model. Nevertheless, further validation in transgenic AD models such as APP/PS1 or 3xTg-AD mice is necessary to gauge its therapeutic relevance to the full spectrum of AD pathology.

The utilization of a multifaceted behavioral test, each of which probes a different cognitive area and brain circuit, is what makes our study strong. LLM consistently reverses SCP-induced deficits in all of these tests, including recognition memory (Novel Object Recognition), short-term working memory (Y-Maze), associative fear memory (Passive Avoidance), and spatial reference memory (Morris Water Maze), indicating a broad-spectrum pro-cognitive effect. This suggests that LLM improves function throughout a network that includes the hippocampus, prefrontal cortex, amygdala, and perirhinal cortex rather than operating only in one area of the brain. The mechanism of LLM may be closely related to cholinergic neurotransmission potentiation, as evidenced by the dose-dependent response and the significant synergistic impact seen with co-administration of DNP.

Molecular docking technology predicts the binding of a candidate ligand to a target receptor. It helps in identifying the key interactions and estimating the binding affinity to allow laboratory studies to prioritize the ligand subjected to docking^39^^[,40^^[,41^. Then, the MD simulation validates binding by simulating the complex movement over time. Also, it assesses the stability of the interaction and provides atomic-level insight into the binding mechanism^42,43^. By removing weak candidates by computer, they form a powerful in silico framework which greatly reduces time and cost of experimental drug screening.

Findings from our in silico study provide a solid molecular basis for this behavior. Molecular docking studies estimate that LLM binds with the catalytic active site of AChE with a high affinity (− 8.7 kcal/mol), forming H-bonds with the residue TYR A:133, TYR A:337, and GLY A:126. This mode of binding was confirmed and explained by 100 ns MD simulations. The low RMSD and RMSF values of LLM in the AChE complex indicates that LLM would be a genuine AchEI, and that may also explain the stability of the complex in the catalytic anionic subsite (CAS) residues (TRP86, PHE295, TYR337, HIS447). The MM-PBSA result showed a negative ΔG_bind which indicates a spontaneous interaction between the studied compounds and indicated strong binding affinity. The energy decomposition analysis revealed that van der Waals forces play a dominant role in binding. It is a common scenario for ligands that interact with the hydrophobic gorge of AChE. While our study about in silico give a probable molecular basis for AChE inhibition, our findings need proper context. As documented in the literature, LLM occurs to be an effective immunomodulatory and anti-inflammatory agent, capable of downregulating the production of pro-inflammatory cytokines TNF-α, IL-6 and IL-8^14,17^. Considering that neuroinflammation is a key contributor of cognitive decline in AD, it is conceivable that the anti-inflammatory effects of LLM could complement its thought to be cholinergic effect. Although we did not measure inflammatory markers in the present study, this is a hypothesis which can be made due to LLM’s known pharmacological actions. Consequently, while AChE inhibition may be the more direct mechanism of action behind the cognitive enhancement we see here, we cannot rule out any inflammatory contribution. Future studies should investigate this.

**Fig. 10:**
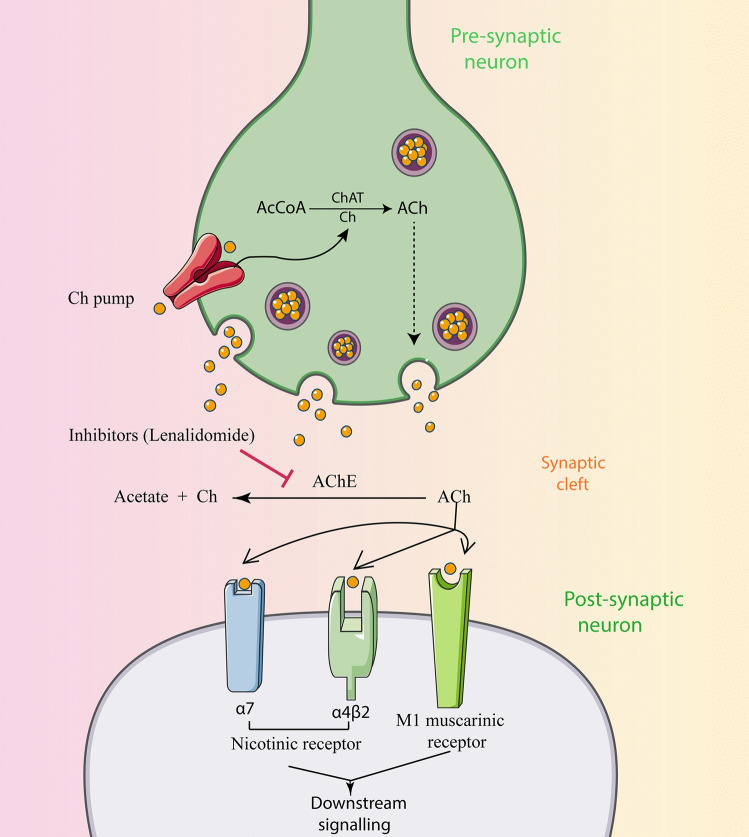
Putative mechanism of lenalidomide-mediated AChE inhibition at cholinergic synapses. LLM inhibits AChE, reducing hydrolysis of ACh and increasing its synaptic availability. Enhanced ACh binding to muscarinic (M1) and nicotinic (α7, α4β2) receptors restores cholinergic signaling, countering scopolamine-induced cognitive deficits. [ACh: Acetylcholine; AChE: Acetylcholinesterase; Ch: Choline; LLM: Lenalidomide].

While our data strongly support AChE inhibition may as a primary mechanism (Fig. 10), LLM’s well-documented pharmacodynamic profile as an immunomodulatory agent suggests potential additional benefits beyond cholinergic enhancement. It is known to downregulate key pro-inflammatory cytokines such as TNF-α, IL-6, and IL-8^14^^[,15^. Given that neuroinflammation is a critical driver of Alzheimer’s pathogenesis, contributing to synaptic dysfunction and neuronal death, it is plausible that LLM’s anti-inflammatory properties contributed to the observed neuroprotection^44^. Furthermore, the synergistic behavioral improvement observed in the LLM + DNP group likely arises from complementary mechanisms: our docking and MD simulations indicate that LLM and DNP share overlapping but non-identical interaction profiles within the AChE gorge. DNP predominantly interacts with PAS residues (e.g., TRP286), whereas LLM forms stable hydrogen bonds near the catalytic anionic site (e.g., TYR337). This suggests potential complementary or allosteric modulation that could enhance overall enzyme inhibition. Thus, the synergy between LLM and DNP may reflect both partially additive AChE inhibition and potential non-cholinergic benefits stemming from LLM’s immunomodulatory profile.

PK and toxicity prediction are critically important in drug discovery, as they determine the in vivo efficacy and safety of a candidate drug, thereby reducing late-stage attrition rates^45–47^. Integrating these studies early in the pipeline allows for the prioritization of compounds with a higher probability of clinical success, saving significant time and resources^48^.

From a drug development perspective, our in silico ADMET predictions using SwissADME and ProTox-3 reveal a promising profile. LLM complies with Lipinski’s Rule of Five, predicts high gastrointestinal absorption, and exhibits a cleaner drug-drug interaction profile than DNP, showing no inhibition of major cytochrome P450 enzymes. Notably, its predicted toxicity profile is more favorable, with a higher LD₅₀ and no predicted activity in carcinogenicity, cytotoxicity, or immunotoxicity endpoints where DNP was flagged. A potential consideration is LLM’s moderately lower predicted CNS permeability (log PS = − 3.074) compared to DNP (log PS = − 1.464). However, LLM has been reported to exhibit favorable brain penetration in preclinical settings^18^, and its significant in vivo cognitive effects observed in this study suggest that sufficient central exposure is achieved. Future pharmacokinetic studies measuring brain concentrations of LLM will help clarify its CNS distribution profile.

While the findings are promising, some limitations of the study should be noted. The acute SCP model does not possess the chronic AD pathology features. The study was sufficiently powered for large effects (n = 5/group). Subtle or long-term outcomes remain unassessed. There is a need for direct biochemical measurements, for example AChE activity or cytokine levels. All together, the integrated in silico and in vivo findings position LLM as a promising multi-target candidate for AD mostly through putative AChE inhibition possibly enhanced by its well-established anti-inflammatory profile. The clues highlighted LLM’s synergistic interaction with DNP, good pharmacokinetic profile and preliminary safety data showing that LLM could be a stand-alone therapeutic or a multi-pharmacology agent. Going forward, it is recommended that future work should include in vitro/ex vivo AChE inhibition assessments, quantification of neuroinflammatory markers, evaluations in transgenic AD models, and development of formulation strategies (for example, nano-formulations or prodrug approaches) to augment CNS delivery and further its therapeutic potential.

## Conclusion

According to the findings of the study, LLM ameliorates cognitive dysfunction in an SCP mouse model, presumably through the mechanism of multi-targeting with possible AChE inhibition and known anti-inflammatory properties. The study of the behavior in vivo indicated that there was a memory and learning effect that is dose dependent and the effect of the high dose (20 mg/kg) was similar to DNP. The combination of LLM and DNP showed synergistic effects indicating increased efficacy for therapy. The in silico analysis established a firm molecular basis for these observations as LLM is a stable binder of the AChE enzyme having a good binding affinity with promising PK and safety properties. We find that the cognitive enhancement of LLM is mediated mainly through the inhibition of AChE, increasing cholinergic transmission. The ability to affect one’s immunity is just one of the facets of the cannabinoids. Consequently, LLM is a potential multi-target therapeutic candidate for AD, which warrants further investigation of its mechanisms and efficacy in chronic, transgenic models.

## Data Availability

Data will be available from the authors on request.
